# A Peek into Their Mind? An Exploration of Links Between Offense-Supportive Statements and Behaviors among Men Who Sexually Exploit Children and Adolescents Online

**DOI:** 10.1177/0306624X211013523

**Published:** 2021-05-06

**Authors:** Sarah Paquette, Francis Fortin

**Affiliations:** 1 Laval University, Quebec, QC, Canada; 2 Internet Child Exploitation Unit, Sûreté du Québec; 3 Université de Montréal, QC, Canada

**Keywords:** criminogenic cognition, implicit theory, child sexual exploitation material, child sexual solicitation, PRESEL

## Abstract

While forensic psychologists have some access to their patients’ thoughts when deciding on a diagnosis or appraising risk, others, such as police investigators, must rely on physical evidence and behavioral markers to make sense of a crime. Studies showing that offense-supportive cognitions constitute a risk factor for sexual offending, including offenses that take place on the internet, highlight the need for some access to offenders’ thoughts. This exploratory study examines the associations between offense-supportive statements about the sexual exploitation of children and adolescents and proxy behaviors. As part of PRESEL, a collaborative research project between Québec provincial police and academic researchers, the case files of 137 men convicted of using child sexual exploitation material or committing child-luring offenses were analyzed. Results showed that many meaningful risk factors and sexual offending behavioral markers were associated with the cognitive themes *Sexualization of children, Child as partner, Dangerous world, Entitlement*, and *Uncontrollability*. The use of encryption was negatively associated with the cognition *Virtual is not real* while *Internet is uncontrollable* was associated with fewer contacts with minors over the internet. Findings are useful for understanding the psychological needs that should be targeted in treatment, as well as helping prioritize police workloads.

The concept of offense-supportive cognitions has received a great deal of attention over past decades. However, despite theoretical and empirical advances, the nature and role of such cognitions in the sexual offending process remain unclear. While there is some evidence to support a link between endorsing specific cognitions and engaging in sexual offending behaviors (Helmus et al., 2013; Paquette & Cortoni, 2019), other researchers have been unable to confirm this finding (Hanson & Morton-Bourgon, 2005; Paquette et al., 2020). Inconsistent conceptualization of the constructs and difficulty in discriminating between post-hoc justifications and criminogenic beliefs may explain these mixed findings (Gannon et al., 2007; Maruna & Mann, 2006). In order to get a sense of the role of cognitions in sexual offending, a first step is to clarify their conceptualization. To do so, various research initiatives have been adopted, one of which uses a thematic analysis of offense-supportive statements to identify underlying themes—understood as *implicit theories*—that are theorized to reflect offender beliefs. Such analysis helps refine our understanding of possible underlying cognitions but provides no certainty that these are actually reflective of what offenders think (see Paquette et al., 2020). However, if certain cognitions are criminogenic, as evidenced by their associations with offending behaviors, then we should be able to use tangible indicators to measure them. In this study, we attempt to determine whether observable markers can help us learn about the criminogenic cognitions of those who commit online and offline sexual offenses against children and adolescents by examining the behavioral correlates associated with particular themes identified within offenders’ discourse.

## Overview of Problems Associated with the Notion of Offense-Supportive Cognitions

Ward (2000) proposed implicit theories as a conceptual framework for understanding the wide and varied range of cognitive distortions observed in the discourse of those who have committed sexual offenses. At the time, the main criticisms of studies of cognitive distortions were an absence of consensus as to how the concept of cognitive distortion should be conceptualized (Gannon et al., 2007) and a lack of understanding of the cognitive process that results in distorted cognitive products—offense-supportive statements. Drawing on cognitive psychology, Ward (2000) suggested that offenders’ descriptions and justifications of their sexual offenses are the result of maladaptive cognitive schemas—*implicit theories*—that are the basis for their views of themselves, their victims, and the world. Implicit theories, like other theories, are used to help individuals “explain empirical regularities (e.g., other people’s actions) and to make predictions about the world” (Ward, 2000, p. 492). In other words, offenders’ behaviors are guided by the beliefs they hold. An example of an implicit theory is the idea that children are sexual beings willing to have sex with adults (Ward & Keenan, 1999).

Several empirical studies have been used to validate Ward’s model and explore whether additional themes were present in various subgroups of sexual offenders (e.g., Bartels & Merdian, 2016; Beech et al., 2005; Beech et al., 2009; Mannix et al., 2013; Marziano et al., 2006; Paquette et al., 2014; Paquette & Cortoni, 2020a; Polaschek & Ward, 2002). Several cognitive themes were found in the discourse of men who sexually offend against minors, whether offline (contact sexual offenders) or online (those who use child sexual exploitation material (CSEM) or solicit sex with children (child sexual solicitors or CSS), that are indicative of their views of children and adolescents, interpersonal relationships, and the virtual environment (Bartels & Merdian, 2016; Howitt & Sheldon, 2007; Paquette & Cortoni, 2020a). However, over two decades after Ward’s initial work, one criticism still remains: our inability to determine with certainty whether offense-supportive statements made by offenders represent deep criminogenic beliefs or are post-hoc justifications formulated to preserve self-image and avoid negative consequences (Ó Ciardha et al., 2017). This criticism becomes even more important when it is recognized that the majority of studies were conducted in settings where offenders could benefit from modulating and dissociating their discourse from their core beliefs (e.g., police interrogations, during incarceration, or in therapeutic follow-up). Alternatively, it is possible that post-hoc justifications, at least some of them, could be both self-preserving and have a criminogenic base. Given the difficulty in accessing the thoughts of offenders, we examine tangible indicators, such as observable behaviors, to provide information about the nature of offenders’ offense-supportive statements. Using tangible evidence and observable behaviors from *in situ* observation would also help prevent the introduction of researcher biases. To achieve this goal, the cognitive themes identified in the literature—whether conceptualized as cognitive distortions, implicit theories, or offense-supportive statements—must be thoroughly understood.

## Unraveling the Key Concepts Underlying the Implicit Theories of Men who Sexually Offend Against Minors

A number of implicit theories have been proposed to reflect the offense-supportive statements made by men who engage in contact sexual abuse of children, including *Children as sexual beings, Dangerous world, Nature of harm, Uncontrollability, Entitlement*, and *Child as partner* (Marziano et al., 2006; Paquette et al., 2014; Ward & Keenan, 1999). These themes have also been found in the discourse of CSEM users and CSS, as have two internet-specific theories: *Virtual is not real*, and *Internet is uncontrollable* (Paquette & Cortoni, 2020a). Other implicit theories, cognitive themes, or variants have been identified in the literature. Despite the common understanding that the cognitive themes represent separate and independent concepts (Paquette et al., 2020), many of them actually overlap in one or many points (Ildeniz & Ó Ciardha, 2019). The following section outlines the theorized and empirically supported conceptualizations of cognitive themes among online and contact sexual offenders against minors.

**Children as sexual beings** refers to the idea that children are either sexual beings or objects as alternatively conceptualized as *Child as sexual being* (Paquette et al., 2014; Paquette & Cortoni, 2020a; Ward, 2000) and *Child as sexual object* (Bartels & Merdian, 2016; Ward & Keenan, 1999). Children’s sexualization is at the core of both these implicit theories. The objectification of children is largely aimed at being able to experience sexual gratification while their humanization as sexual actors attributes sexual intentions, desires, and maturity comparable to that of adults to them. Holding either of these implicit theories could thus underpin sexual interests toward children (Bartels & Merdian, 2016; Ildeniz & Ó Ciardha, 2019). This hypothesis has been investigated in several studies. In one, pedophilic men were found to reveal more implicit associations linking children to sexuality than non-pedophilic comparison groups (Brown et al., 2009; Gray et al., 2005). Marziano et al. (2006) found that men who sexually victimized male children tended to endorse the *Children as sexual beings* implicit theory more strongly than those who sexually victimized female children. Howitt and Sheldon (2007) found that CSEM users endorsed higher levels of cognitions supporting sexualization of children than men who sexually abuse children. Using a different conceptualization and methodology, Paquette and her colleagues (2020) found evidence of an association between this cognitive theme and contact sexual offending against children among men who also engage in online sexual exploitation of children. Taken together, these findings suggest that this belief supports pedophiliac interests and involvement of children in sexual activities.

To help alleviate difficulties in measuring pedophilia through self-report questionnaires, Seto and Eke (2017) developed the Correlates of Admitted Sexual Interest in Children (CASIC) scale, which assesses this interest using proxy behavioral indicators. These indicators are (1) never married, (2) possessing child pornography videos or (3) sex stories, (4) consuming such CSEM over a period of 2 or more years, (5) volunteering in a role with high access to children, and (6) engaging in online sexual communication with minors. A high number of child victims can also be considered a behavioral indicator of sexual interest in children (Cantor & McPhail, 2015) and could therefore also serve as a proxy for *Children as sexual beings*.

**Nature of harm** is the belief that sexual acts involving children are harmful to varying degrees—that some sexual acts in which children are involved do not harm them, or at least are not harmful to the same level as others, while some sexual activities might even be pleasant and beneficial (Bartels & Merdian, 2016; Paquette et al., 2014; Paquette & Cortoni, 2020a; Ward & Keenan, 1999). Several authors have suggested or found that this belief is closely related to *Children as sexual beings* (Bartels & Merdian, 2016; Burke, 2011; Ildeniz & Ó Ciardha, 2019; Paquette & Cortoni, 2020a; Ward & Keenan, 1999). We argue that, from a theoretical perspective, no distinction should be made between these two themes as both appear to support pedophilic interest (see also Paquette & Cortoni, 2020a). Both define children’s reality in a self-serving manner that makes it possible to maintain sexual fantasies: in *Children as sexual objects*, the child is dehumanized, reduced to the status of an object; in *Children as sexual beings*, characteristics associated with sexual maturity are attributed to the child. In *Nature of harm*, a selective filter is applied to eliminate evidence of maltreatment of children resulting from sexual activities. All these interpretations serve to eliminate barriers to actions involving children that lead to sexual gratification. This is consistent with psychological research that shows that various cognitive processes, such as attribution, attitudes, and perceptions, are subject to modulation by self-serving interests (see Kunda, 1990 for a review). In this context, behaviors related to *Nature of harm* would be expected to be the same as those associated with *Children as sexual beings/objects*.

**Child as partner** refers to the belief held by some adult males that they and the children they interact with have the same level of emotional, affective, and social maturity. Children are thus seen as potential friends or partners (Paquette et al., 2014). This implicit theory has been further conceptualized as the cognitive component of a psychological process of self-identification with children known as *emotional congruence with children* (Paquette & McPhail, 2020). This process is not only criminogenic by nature, as evidenced by its association with sexual offending against children (Mann et al., 2010; McPhail et al., 2013), but can also reflect a positive view of children, a preference for relationships with children rather than with adults, a childlike sense of self, and a romantic attraction to children (Paquette & Cortoni, 2020a; Waldron et al., 2006). It has also been found to be related to other psychologically meaningful constructs such as pedophilia and other paraphilias, immaturity, difficulties in sexual coping, and sexual preoccupation (Hermann et al., 2017; McPhail et al., 2014, 2018; Paquette & McPhail, 2020).

Some men who demonstrate cognitive and emotional congruence with children may adopt child-related or childlike activities. Behavioral indicators include, among other things, frequent and close contact with children in both sexual and non-sexual contexts, such as having a job or engaging in volunteering that requires contact with minors (e.g., scout leader, bus driver, school teacher, baseball coach) or having frequent sexual relationships with minors (Knight et al., 1989). There is also evidence of an association between possessing texts that involve children and sexual interest in children (Seto & Eke, 2017). We suggest that the possession of romantic child pornography texts is associated with the cognitive theme *Child as partner*.

**Dangerous world** was first conceptualized to capture the belief that the world is a dangerous place in which people behave in abusive and rejecting ways (Polaschek & Ward, 2002; Ward & Keenan, 1999). This view of the world includes a need to punish, dominate, or take revenge on those who are seen as intending harm. Adults, particularly women, are perceived as abusive, hostile, rejecting, threatening, and controlling (Bartels & Merdian, 2016; Ildeniz & Ó Ciardha, 2019; Paquette et al., 2014; Paquette & Cortoni, 2020a; Ward & Keenan, 1999). It has been argued that fear and anger toward adults contribute to this distorted view of the world, a suggestion that has received empirical support in the association between endorsing cognitions supportive of a dangerous world theory and being hostile toward others, particularly toward women (Ildeniz & Ó Ciardha, 2019).

A variant of this belief is the *Unhappy world* implicit theory proposed by Bartels and Merdian (2016) to describe how men who use CSEM tend to see the world as limiting and unsatisfying. They stress that a negative and melancholy perception of the world could result in loneliness and a difficulty with engaging in and maintaining close and meaningful intimate relationships with others. Supporting this theory, problematic relationships and indications of a lack of intimacy were found in a study of the discourse of CSEM users (Rimer, 2016). Findings about both CSEM users and those who solicit minors for sexual purposes suggest that both virtual and offline relationships are affected by a negative and fearful perception of others (Paquette & Cortoni, 2020a). Other related problems might also be associated with this way of thinking, including emotional and relational problems (e.g., social avoidance, fearful attachment style; Bartels & Merdian, 2016). Burke (2011) found a link between the cognitive theme *Dangerous world* and having experienced childhood abuse, which could partly explain why some men mistrust interpersonal relationships. Marziano and his colleagues (2006) observed that those who had experienced childhood sexual victimization endorsed significantly higher levels of this cognitive theme than those who had not.

Another aspect of this implicit theory is that children, unlike adults, are seen as safe, reliable, and trustworthy (Ildeniz & Ó Ciardha, 2019; Paquette et al., 2014; Paquette & Cortoni, 2020a; Ward, 2000; Ward & Keenan, 1999) and relationships with children are therefore more satisfying than those with adults (Paquette & Cortoni, 2020a). According to Ildeniz and Ó Ciardha (2019), this perception is closely related to feeling an emotional congruence with children, as children are perceived to be more accepting and capable of unconditional love than adults. The authors further suggest that this theory “involves the perception that children are capable of understanding the oﬀender’s needs and sexual desires and are willing to gratify them” (p. 830), which is closely related to *Children as sexual beings*. Paquette (2018, p. 209, authors’ translation) hypothesized that “if an offender believes that the world is a dangerous place, this may affect his relationships with other adults, resulting in a multitude of problems related to intimacy and sexuality, including emotional congruence with children and distant attachment styles.” While the association between *Dangerous world* and problematic relationships has not yet been empirically tested, Ildeniz and Ó Ciardha (2019) found support for an association between this belief and the constructs emotional congruence with children and *Children as sexual beings*.

**Entitlement** refers to a sense of having the right to act however one pleases. Others are expected to obey commands and fulfill desires because the individual sees himself as having a privileged and special status, a status based on being a man, belonging to a particular social class, or holding a superior position in a couple or family relationship (Paquette et al., 2014; Paquette & Cortoni, 2020a; Ward & Keenan, 1999). The main characteristic of men who exhibit such sentiments is a total lack of consideration for others, which translates into a refusal to acknowledge moral and legal rules (i.e., the right to commit acts prohibited by law, refusal to acknowledge the needs and desires of others). Paquette et al. (2020) found that a sense of entitlement was associated with involvement in crime as evidenced by a higher number of criminal charges and more diverse criminal behavior. Given this, as well as the fact that criminal involvement is also associated with *Dangerous world*, including a need to achieve dominance and control over others, we suggest that these cognitive themes may underlie the criminogenic cognitions that allow offenders to act on their antisocial tendencies.

**Uncontrollability** relates to the perception that the world is inherently uncontrollable and that some individuals, due to internal and external factors, are unable to control their desire to engage in sexual exploitative behaviors with children and adolescents (Bartels & Merdian, 2016; Paquette et al., 2014; Paquette & Cortoni, 2020a; Ward & Keenan, 1999). Internal factors are negative emotions such as depression and stress; being addicted, bored, or lonely; having strong sexual impulses and preoccupations; or being under the influence of uncontrollable religious and spiritual forces. External factors are negative life events such as substance abuse, childhood abuse, job loss, and marital separation (Bartels & Merdian, 2014; Burke, 2011; Elliot, 2011; Paquette et al., 2014; Paquette & Cortoni, 2020a; Ward & Keenan, 1999).

**Virtual is not real** captures the ideas of men who sexually exploit children and adolescents online that the internet’s content is unreliable and not reflective of reality: everything seen or read on the internet is thus assumed to be untrue, fake, or a lie (Paquette & Cortoni, 2020a; Rimer, 2016, 2019). These men tend to feel that there is a clear-cut distinction between the virtual and the offline world and thus children depicted in sexual images are assumed either not to exist or to be only digital composites, while those with whom offenders communicate are assumed to be adults acting as children. According to Rimer (2019), such perceptions—which create the idea that there is no actual contact with children—emphasize the distance between the individual and any abusive behaviors. This distance may be further reinforced by individuals’ “use of the internet and [its] related distancing, detachment, and anonymity” (Rimer, 2019, p. 167). Bartels and Merdian (2016) suggest that use of the internet has an influence on all cognitions rather than constituting an implicit theory. The authors further highlight “how strongly [implicit theories] are embedded within the context in which they occur” (p. 18). In this context, the properties of the internet could potentially have an effect on any type of behavior.

**Internet is uncontrollable** refers to the idea that the internet is widely accessible in terms of both users and content and facilitates uncontrollable behavior (Paquette & Cortoni, 2020a). This cognition would thus be influenced by the properties of the internet. In this context, we argue that the *Internet is uncontrollable* should be associated with offenders’ online behaviors, including those related to attempts to remain anonymous.

## Present Study

For researchers and practitioners, gaining access to the thoughts of those who commit sexual crimes is a challenge, especially for those in agencies such as law enforcement where direct access is limited or unavailable. While it is acknowledged that some individuals report offense-supportive statements—and perhaps beliefs—about sexual offending behaviors against children and adolescents, it is important to also be able to assess their cognitions. Many studies have attempted to shed light on the exact nature of offense-supportive cognitions and have identified numerous theoretical and empirical associations between these cognitions and meaningful risk factors and behaviors. In their etiological model of risk, Ward and Beech (2004) argue that dynamic risk factors, including offense-supportive cognitions, “are really the surface representation of underlying traits indicative of deeper causal properties of a person’s functioning” (Cattell & Kline, 1977, p. 274). The way individuals think and speak may thus be influenced by developmental or contextual factors, some of which may be available to practitioners (e.g., information on childhood situation, marital status, criminal records). Outside the field of sexual offending, social and cognitive psychology researchers have found associations between individuals’ cognitions and their behaviors (see Fiske & Taylor, 1991, for a review), thus again suggesting that the way offenders speak about their crimes may be related to some non-offending behaviors and markers. Given this, the current exploratory study is aimed at examining whether there are tangible markers—meaningful risk factors, either sexual or non-sexual, as well as non-offending behaviors—that could help those in law enforcement agencies determine the presence of offense-supportive cognitions that are not expressed or are voluntarily hidden by men who sexually exploit minors.

## Method

### Sample

**Figure 1.**
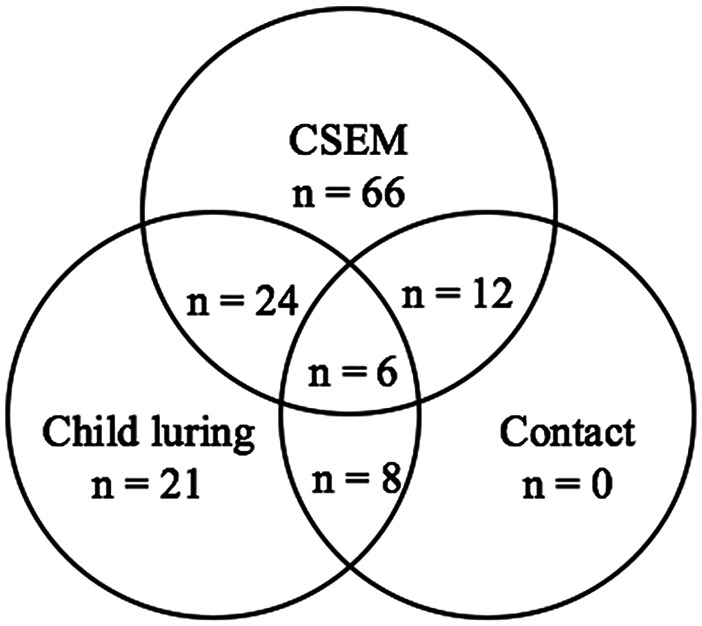
Sample representation of indexed sexual charges.

This study is based on 137 police randomly selected cases between 2006 and 2017 and was part of PRESEL (a French acronym for *Projet de recherche sur l’exploitation sexuelle des enfants en ligne*), a collaborative research project between the Sureté de Police de Québec and academic researchers. The PRESEL project is aimed at gaining a better understanding of men who sexually offend against minors online to aid in the development of efficient intervention strategies and systematic case prioritization tools based on evidence-based risk factors. A wide range of socio-demographic, developmental, interpersonal, and sexo-criminological variables were examined for adult males convicted of child pornography, child luring offenses, or both. Some had also been convicted of contact sexual offenses against minors (see Figure 1). The average age of men in our sample was 39.35 years old (*SD* = 14.74, range = 18–81) and 43.8% (*n* = 60, missing cases = 3) were engaged in an intimate relationship at the time of their index crime. Subgroups of offenders were 66 men convicted of CSEM only, 21 of child luring offenses only, 24 of mixed online sexual offenses (both CSEM and child luring), and 26 of mixed online and offline sexual offenses against children and adolescents (i.e., either or both CSEM and child luring as well as contact sexual offenses).

### Ethics

The study received ethical approval from both the university and the law enforcement agency involved in the project.

### Procedure

As part of the PRESEL project, a coding book was created by the first author. The way variables were conceptualized was reviewed by both the second author and an external expert in the field of forensic psychology. Variables were defined based on a wide range of scientific literature (e.g., Mann et al. 2010 for meaningful risk factors and Paquette & Cortoni 2020a for cognitive themes) and examples were provided to support the codification. Research assistants were trained in the use of the coding book by the first author. Police interrogations were part of the material available for codification and, given that many of them lasted a long time (up to 10 hours in some cases, and 4–5 hours on average), a subsample (*n* = 9 cases) was coded by two research assistants for inter-judgment reliability. The overall agreement in the coding of variables reached 84%. Individual item definitions, related examples, and inter-rater agreement are shown in Table S1.

### Measures

Based on the scientific literature review as well as information from police cases, the following variables were coded and organized into four categories:

#### Cognitive themes

Themes were created based on existing literature and on the conceptualizations of offense-supportive statements as cognitive themes identified in the discourse of online and contact sexual offenders against children (Paquette & Cortoni, 2020a; Paquette et al., 2014, 2020). *Sexualization of children*, a composite of *Child as sexual being* and *Nature of harm*, reflects offenders’ statements supporting the idea that children are sexual beings and that engaging in sexual activities with them does not harm them (coded as present = 1 (30.7%) or absent/no information = 0). *Child as partner* refers to offenders’ perception that children can be intimate and life partners (coded as present = 1 (8.0%) or absent/no information = 0). *Dangerous world* reflects offenders’ statements supporting the idea that the world and adults are dangerous and threatening (coded as present = 1 (7.3%) or absent/no information = 0). *Entitlement* refers to offenders’ statements suggesting that they are entitled to act as they please, even in sexual interactions with children (coded as present = 1 (7.3%) or absent/no information = 0). *Uncontrollability* reflects the discourse of offenders who place responsibility for their offending behaviors on uncontrollable internal or external factors (coded as present = 1 (26.3%) or absent/no information = 0). *Virtual is not real* refers to statements supporting the idea that internet and virtual contents do not represent reality (coded as present = 1 (40.1%) or absent/no information = 0). *Internet is uncontrollable* suggests that because the internet provides easy access to many users and content, online sexual offending cannot be controlled (coded as present = 1 (36.5%) or absent/no information = 0). See Table S1 for full descriptions and examples of these cognitive themes.

#### Meaningful risk factors

The following variables were considered because of their empirically supported, or promising, associations with sexual offending behaviors (see Mann et al., 2010 for an exhaustive review): pedophilia (either self-reported sexual interest in children or formally diagnosed; coded as present = 1 (38%) or absent/no information = 0); sexual strategies used to cope with negative moods (masturbation, fantasies, pornography; *x̄* = .18, *SD* = .53, range = 0–3); self-reported sexual preoccupation (present = 1 (20.4%) or absent/no information = 0); self-reported sexual preference for boys (preference for boys = 1 (15.3%) or preference for girls/no preference/no information = 0); self-reported emotional congruence with children (present = 1 (11.7%) or absent/no information = 0); never in an intimate relationship (present = 1 (22.6%) or absent/no information = 0); length (in months) of the longest relationship (*x̄ =* 127.43, *SD* = 139.90, range = 0–612); self-reported relational difficulties (present = 1 (26.3%) or absent/no information = 0); abuse during childhood (present = 1 (29.9%) or absent/no information = 0); CASIC total score (*x̄ =* 1.85, *SD* = 1.06, range = 0–5); and two recidivism measures, the CPORT (*x̄ =* 2.08, *SD* = 1.30, range = 0–6) and the STATIC-99 (*x̄ =* 2.61, *SD* = 1.59, range = 1–9).

#### Non-offending behaviors

To take into account previous theoretical or empirical associations with one or more of the cognitive themes, the following non-offending behaviors were examined: physical access to minors through volunteering activities (present = 1 (15.3%) or absent/no information = 0); physical access to minors through work (present = 1 (10.2%) or absent/no information = 0); physical access to minors through the immediate family (present = 1 (45.3%) or absent/no information = 0); virtual access to minors through online networks (present = 1 (48.9%) or absent/no information = 0); number of different types of access to minors (*x̄ =* 1.61, *SD* = .90, range = 0–5); use of encryption while engaging in online crime (present = 1 (12.4%) or absent/no information = 0); use of proxy or TOR browser while engaging in online crime (present = 1 (1.5%) or absent/no information = 0); use of public computers during the index crime (present = 1 (14.6%) or absent/no information = 0); number of different strategies used to preserve anonymity (i.e., encryption, proxy/TOR, public computers; *x̄ =* .40, *SD* = .587, range = 0–2); self-reported feelings of boredom (present = 1 (10.2%) or absent/no information = 0); self-reported feelings of loneliness (present = 1 (26.3%) or absent/no information = 0).

#### Sexual and non-sexual behaviors

The following indicators of sexual and non-sexual offending behaviors were examined: number of known contact child victim (*n* = 39, *x̄ =* 2.51, *SD* = 2.29, range = 1–12); number of male child victims (*x̄* = .92, *SD* = 2.10, range = 0–12); number of child pornography images collected (*x̄ =* 20,330.99, *SD* = 118,555.94, range = 0–1,071,226), lifetime number of contacts sexual charges against children (*x̄* = .71, *SD* = 1.77, range = 0–11); lifetime number of child pornography charges (*x̄ =* 2.82, *SD* = 4.01, range = 0–38); lifetime number of child luring charges (*x̄ =* 1.79, *SD* = 4.42, range = 0–34); self-reported masturbation while viewing minors (either CSEM or live via webcam; present = 1 (24.8%) or absent/no information = 0); possession of romantic child pornography texts (present = 1 (3.6%) or absent/no information = 0); self-reported abuse of drugs or alcohol (present = 1 (19.0%) or absent/no information = 0); childhood criminal history (present = 1 (5.8%) or absent/no information = 0); length (in days) of sentence for index crime (*x̄ =* 450.29, *SD* = 400.77, range = 0–2373); lifetime number of charges for violent offenses (*x̄ =* .33, *SD* = 1.35, range = 0–14); lifetime number of charges for breach of conditions (*x̄ =* 1.14, *SD* = 2.38, range = 0–13); lifetime number of charges for general non-sexual non-violence offenses (*x̄ =* 1.71, *SD* = 4.03, range = 0–19); diversity of criminal behaviors (i.e., sexual, violent, general; *x̄ =* 1.69, *SD* = .67, range = 1–3).

### Analyses

Subgroups of offenders were compared with each of the cognitive themes in order to determine whether their presence was significantly associated with a specific type of sexual offending behavior involving children and adolescents. Pearson’s correlations were then conducted to examine associations between the variables and cognitive themes. Finally, individual Receiver Operating Characteristic (ROC) curve analysis was conducted for each cognitive theme. By convention, results were interpreted as follows: area under the curve (AUC) of 1 means the variable perfectly distinguished those with the criterion (i.e., the presence of a specific cognitive theme) from those who without it, while .5 means that the variable did not discriminate better than chance. AUCs of .54, .63, and .71 are considered small, moderate, and large effect sizes, respectively (Rice & Harris, 2005).

## Results

Cognitive themes were observed in all subgroups of sexual offenders against children and adolescents. As can be seen in Table 1, omnibus chi-square tests were significant for both *Sexualization of children* and *Internet is uncontrollable*, but a significant group difference was found only in CSEM users for the *Internet is uncontrollable*.

**Table 1.** Presence (%) of the Cognitive Themes in Subgroups of Sexual Offenders.

	CSEM users only *n* = 66 (%)	CL only *n* = 21 (%)	Online mixed *n* = 24	Contact mixed *n* = 26 (%)	***X***^2^ (*df*), Phi
Sexualization of children	21 (31.82)	5 (23.81)	3 (12.50)	13 (50.00)	8.80* (3), .25
Child as partner	4 (6.06)	1 (4.76)	3 (12.50)	3 (11.54)	1.73 (3), 11
Dangerous world	7 (10.61)	0 (0)	0 (0)	3 (11.54)	5.30 (3), .20
Entitlement	4 (6.06)	3 (14.29)	0 (0)	3 (11.54)	4.25 (3), .18
Uncontrollability	19 (28.79)	4 (19.05)	5 (20.83)	8 (30.77)	1.42 (3), .102
Virtual is not real	24 (36.92)	10 (47.62)	10 (41.67)	11 (42.31)	0.84 (3), .08
Internet is uncontrollable	32 (48.49)^a^	6 (28.57)	3 (12.50)	9 (34.62)	10.66* (3), .28

*Note*. CSEM = child sexual exploitation material; CL = child luring.

* ≤.05.

a Chi-square post-hoc test using adjusted residuals indicate a *p*-value significantly lower than the Bonferroni corrected *p*-value.

Results from the associations between meaningful risk factors and cognitive themes are shown in Table 2. *Sexualization of children* was positively associated with sexual interest toward children (*r =* .27; AUC = .63, *p* < .05), sexual preoccupation (*r* = .17, *p* < .05), sexual preference for boys (*r =* .24, *p* < .05), sex as coping strategies (*r* = .17, *p* < .05), CASIC (*r* = .17, *p* < .05) and CPORT (*r* = .17, *p* < .05) scales. *Child as partner* was positively associated with sexual interest toward children (*r =* .26; AUC = .74, *p* < .01), sexual preference for boys (*r =* .24, *p* < .05), emotional congruence with children (*r =* .50; AUC = .78, *p* < .01), history of childhood abuse (*r =* .22; AUC = .68, *p* < .05), CASIC (*r* = .22; AUC = .75, *p* < .01) and CPORT (*r* = .19, *p* < .05) scales. *Dangerous world* was positively associated with emotional congruence with children (*r* = .21, *p* < .05), relational difficulties (*r* = .25; AUC = .79, *p* < .05), never been in an intimate relationship (*r* = .30; AUC = .76, *p* < .01), history of childhood abuse (*r* = .25; AUC = .72, *p* < .01) and negatively associated with the length of the longest intimate relationship (*r* = −.25; AUC = .82, *p* < .01). *Entitlement* was positively associated with sexual preoccupation (*r* = .21, *p* < .05) and STATIC99 (*r* = .19, *p* < .05). *Uncontrollability* was positively associated with the CPORT scale (*r* = .17, *p* < .05) and negatively with the length of the longest intimate relationship (*r* = −.24, *p* < .05). *Virtual is not real* was negatively associated with CASIC (*r* = −.08, *p* < .05) and STATIC99 (*r* = −.25; AUC = .62, *p* < .05) scales. *Internet is uncontrollable* was negatively associated with relational difficulties (AUC = .70, *p* < .05).

**Table 2.** Meaningful Risk Factors: Correlations and Predictors of Cognitive Themes.

	Sexualization of children	Child as partner	Dangerous world	Entitlement	Uncontrollability	Virtual is not real	Internet is uncontrollable
	*r*	*r*	*r*	*r*	*r*	*r*	*r*
	AUC [95% CI]	AUC [95% CI]	AUC [95% CI]	AUC [95% CI]	AUC [95% CI]	AUC [95% CI]	AUC [95% CI]
Sexual interest toward children	.27**	.26**	−.01	.14	.12	.03	.03
	.63* [.53, .74]	.74** [.60, .89]	.50 [.32, .68]	.63 [.35, .91]	.56 [.36, .77]	.51 [.37, .64]	.53 [.42, .64]
Sexual preoccupation	.17*	.05	−.00	.21*	.07	.10	.07
	.58 [.47, .68]	.54 [.35, .72]	.50 [.31, .69]	.60 [.32, .88]	.53 [.42, .64]	.54 [.41, .68]	.52 [.41, .63]
Sexual preference for boys	.24*	.24*	.03	.01	.06	−.12	−.03
	.60 [.48, .72]	.66 [.47, .86]	.52 [.31, .74]	.50 [.23, .76]	.54 [.34, .74]	.51 [.35, .66]	.45 [.27, .62]
Sex as coping strategies	.17*	.06	.01	.01	−.04	.01	.09
	.54 [.43, .65]	.54 [.35, .72]	.49 [.31, .68]	.44 [.20, .69]	.52 [.38, .67]	.51 [.38, .64]	.52 [.42, .62]
Emotional congruence with children	.05	.50**	.21*	.13	.00	.06	−.04
	.52 [.42, .63]	.78** [.61, .96]	.64 [.44, .84]	.55 [.27, .84]	.46 [.27, .66]	.56 [.43, .70]	.54 [.37, .72]
Relational difficulties	−.03	.14	.25*	.03	.06	−.19	−.16
	.52 [.38, .65]	.66 [.40, .92]	.79* [.65, .93]	.57 [.31, .83]	.53 [.39, .68]	.60 [.45, .76]	.70* [.54, .86]
Never been in an intimate relationship	.06	.12	.30**	.12	.14	−.17	.07
	.53 [.42, .64]	.60 [.40, .80]	.76* [.58, .94]	.48 [.22, .74]	.49 [.29, .69]	.61 [.46, .76]	.53 [.42, .64]
Length of the longest intimate relationship	.15	−.15	−.25*	−.06	−.24*	.10	−.03
	.54 [.39, .69]	.65 [.46, .84]	.82** [.69, .94]	.53 [.32, .74]	.63 [.50, .76]	.60 [.47, .73]	.44 [.26, .62]
History of childhood abuse	.08	.22*	.25**	−.06	.05	−.02	−.13
	.54 [.44, .65]	.68* [.51, .86]	.72* [.55, .89]	.52 [.28, .76]	.57 [.38, .76]	.43 [.28, .59]	.56 [.46, .66]
CASIC	.17*	.22**	.12	.07	.04	−.08	.08
	.58 [.48, .69]	.74** [.60, .87]	.65 [.49, .81]	.40 [.15, .65]	.55 [.36, .75]	.68* [.53, .82]	.54 [.43, .65]
CPORT	.31**	.15	.00	.09	.17*	−.10	.01
	.68** [.57, .78]	.66 [.53, .78]	.55 [.42, .67]	.60 [.43, .77]	.73* [.57, .90]	.54 [.38, .70]	.52 [.41, .63]
STATIC99	.13	.19*	−.02	.19*	.15	−.25**	−.13
	.58 [.48, .69]	.67 [.51, .83]	.50 [.31, .69]	.66 [.46, .85]	.68 [.49, .85]	.62* [.53, .71]	.67 [.51, .84]

*Note*. Predictors in negative correlations were inverse for ROC analyses.

* *p* < .05; **p < .01.

Results from the associations between non-offending behaviors and cognitive themes are shown in Table 3. *Sexualization of children* was positively associated with access to minors via volunteering (*r* = .20, *p* < .05) and negatively associated with access to minors via online networks (*r* = −.18, *p* < .05). *Child as partner* was positively associated with access to minors via volunteering (*r* = .17, *p* < .05) and the number of modes of access (*r* = .19, *p* < .05). *Virtual is not real* was positively associated with access to minors via online networks (*r* = .18, *p* < .05) and the number of modes of access (*r* = .22, *p* < .05), and negatively associated with the use of encryption (*r* = −.32; AUC = .63, *p* < .05). *Internet is uncontrollable* was negatively associated with access to minors via online networks (*r* = −.32, AUC = .67, *p* < .01).

**Table 3.** Non-Offending Behaviors: Correlations and Predictors of Cognitive Themes.

	Sexualization of children	Child as partner	Dangerous world	Entitlement	Uncontrollability	Virtual is not real	Internet is uncontrollable
	*r*	*r*	*r*	*r*	*r*	*r*	*r*
	AUC [95% CI]	AUC [95% CI]	AUC [95% CI]	AUC [95% CI]	AUC [95% CI]	AUC [95% CI]	AUC [95% CI]
Access to minors via volunteering	.20*	.17*	.11	.04	−.07	.02	.10
	.63 [.26, .99]	.61 [.42, .81]	.46 [.00, 1.00]	.44 [.11, .78]	.40 [.06, .74]	.43 [.11, .75]	.58 [.26, .91]
Access to minors via work	.09	.17	−.10	−.00	.07	.07	.05
	.75 [.41, 1.00]	.59 [.40, .81]	.58 [.05, 1.00]	.43 [.07, .79]	.70 [.38, 1.00]	.36 [.05, .67]	.51 [.19, .84]
Access to minors via family	.06	.11	−.09	.08	.02	.16	.10
	.53 [.18, .88]	.60 [.43, .77]	.21 [.00, .53]	.53 [.18, .88]	.45 [.12, .78]	.54 [.21, .86]	.38 [.06, .70]
Access to minors via online networks	−.18*	.03	−.11	.01	−.02	.18*	−.32**
	.58 [.23, .94]	.53 [.35, .71]	.83 [.56, 1.00]	.42 [.07, .77]	.68 [.36, .99]	.55 [.22, .87]	.67** [.57, .76]
Number of modes of access	.16	.19*	−.13	.15	−.00	.22*	−.05
	.68 [.29, 1.00]	.64 [.46, .82]	.46 [.04, .88]	.54 [.17, .91]	.46 [.07, .86]	.44 [.11, .77]	.57 [.24, .90]
Encryption	.08	.09	−.10	.02	−.02	−.32**	−.10
	.63 [.26, .99]	.59 [.22, .95]	.54 [.00, 1.00]	.63 [.26, .99]	.56 [.24, .89]	.63* [.51, .75]	.54 [.41, .66]
Hidden identity software	.06	−.05	−.04	−.06	−.10	−.17	−.16
	.50 [.15, .85]	.51 [.25, .77]	.50 [.00, 1.00]	.50 [.15, .85]	.50 [.17, .84]	.53 [.39, .67]	.53 [.40, .66]
Public computer	.04	−.07	−.03	−.06	−.17	.03	−.05
	.28 [.00, .56]	.47 [.20, .74]	.13 [.00, .36]	.54 [.19, .89]	.75 [.48, 1.00]	.52 [.20, .85]	.52 [.41, .63]
Number of anonymity strategies	.14	.12	−.15	−.05	−.13	−.06	.08
	.53 [.18, .88]	.83 [.63, 1.00]	.21 [.00, .53]	.47 [.12, .83]	.71 [.42, 1.00]	.53 [.43, .62]	.54 [.44, .64]
Feeling bored	−.14	−.29	.23	−.08	.14	.07	−.07
	.71 [.39, 1.00]	.83 [.59, 1.00]	.75 [.39, 1.00]	.53 [.18, .88]	.55 [.22, .88]	.46 [.14, .79]	.54 [.21, .86]
Feeling lonely	−.11	.21	.21	−.05	−.05	.16	−.13
	.65 [.33, .98]	.66 [.40, .92]	.79 [.47, 1.00]	.47 [.12, .83]	.39 [.06, .72]	.54 [.21, .86]	.62 [.30, .94]

*Note*. Predictors in negative correlations were inverse for ROC analyses.

* *p* < .05; ** *p* < .01.

Results from the associations between sexual and non-sexual offending behaviors and cognitive themes are shown in Table 4. *Sexualization of children* was positively associated with the overall number of contact sexual charges (*r* = .24, *p* < .05), masturbating while viewing minors (*r* = .30, *p* < .05), and the length of the index crime sentence (*r* = .24, *p* < .05). *Child as partner* was positively associated with the number of child pornography images (*r* = .31, *p* < .05) and the possession of CSEM with romantic component (*r* = .58, *p* < .05). *Entitlement* was positively associated with the overall number of contact sexual charges (*r* = .17, *p* < .05), the overall number of contact child luring charges (*r* = .24, *p* < .05), the length of the index crime sentence (*r* = .28; AUC = .72, *p* < .05), the overall number of violent (*r* = .31, *p* < .01) and general (AUC = .69, *p* < .05) charges, as well as with the diversity of the criminal behaviors (*r* = .30, AUC = .79, *p* < .01). *Uncontrollability* was positively associated with childhood crime history (*r* = .51, *p* < .05) and the total number of breach of conditions charges (*r* = .17, *p* < .05). *Virtual is not real* was negatively associated with the total number of child pornography charges (*r* = −.19, AUC = .61, *p* < .05). *Internet is uncontrollable* was positively associated with the total number of child pornography charges (AUC = .60, *p* < .05) and negatively associated with the number of child luring charges (*r* = −.21, AUC = .70, *p* < .01).

**Table 4.** Sexual and Non-Sexual Offending Behaviors: Correlations and Predictors of Cognitive Themes.

	Sexualization of children	Child as partner	Dangerous world	Entitlement	Uncontrollability	Virtual is not real	Internet is uncontrollable
	*r*	*r*	*r*	*r*	*r*	*r*	*r*
	AUC [95% CI]	AUC [95% CI]	AUC [95% CI]	AUC [95% CI]	AUC [95% CI]	AUC [95% CI]	AUC [95% CI]
Number of child victims	.03	.08	−.02	.15	.23	−.11	−.23
	.48 [.30, .67]	.66 [.44, .89]	.44 [.15, .73]	.64 [.34, .94]	.60 [.38, .81]	.59 [.38, .81]	.62 [.44, .80]
Number of male child victims	.12	.13	−.08	−.02	.25	−.21	−.16
	.50 [.32, .69]	.57 [.24, .89]	.57 [.27, .87]	.49 [.21, .78]	.54 [.31, .77]	.66 [.48, .84]	.54 [.36, .73]
Number of child pornography images	.11	.31**	−.05	−.04	−.08	−.10	.16
	.56 [.44, .69]	.57 [.35, .78]	.44 [.26, .62]	.41 [.12, .69]	.48 [.35, .61]	.59 [.47, .71]	.49 [.36, .61]
Number of contact sexual charges	.24**	.03	−.03	.17*	.11	−.10	−.02
	.60 [.49, .71]	.56 [.38, .75]	.53 [.35, .71]	.60 [.39, .80]	.51 [.40, .62]	.56 [.46, .66]	.53 [.43, .63]
Number of child pornography charges	.03	−.01	.01	−.07	.03	−.19*	.08
	.57 [.47, .68]	.41 [.27, .54]	.59 [.42, .76]	.61 [.43, .79]	.54 [.43, .65]	.61* [.52, .71]	.60* [.51, .70]
Number of child luring charges	−.01	−.03	−.10	.24**	.02	−.01	−.21*
	.56 [.45, .67]	.52 [.33, .70]	.64 [.49, .79]	.59 [.38, .81]	.48 [.37, .59]	.48 [.38, .58]	.70** [.61, .79]
Masturbating while viewing minors	.30*	−.03	.13	.09	−.09	−.06	−.07
	.65 [.49, .81]	.53 [.18, .88]	.58 [.36, .80]	.56 [.33, .80]	.54 [.36, .72]	.53 [.36, .69]	.53 [.36, .70]
Written CSEM with romantic component	.12	.23**	−.06	−.06	−.12	−.08	.10
	.53 [.42, .63]	.58 [.39, .77]	.52 [.34, .70]	.52 [.34, .70]	.53 [.42, .63]	.52 [.42, .61]	.52 [.42, .62]
Substance abuse	.13	.01	.07	.01	.25	−.21	.13
	.57 [.41, .74]	.51 [.21, .80]	.56 [.30, .83]	.51 [.26, .75]	.63 [.48, .79]	.61 [.45, .77]	.57 [.41, .74]
Childhood crime history	.05	.06	−.19	.22	.51*	.08	−.21
	.54 [.28, .80]	.56 [.12, .99]	.71 [.33, 1.00]	.65 [.30, .99]	.84 [.66, 1.00]	.54 [.27, .81]	.61 [.34, .87]
Length of index crime sentence	.24**	.09	−.14	.28**	.08	−.17	−.05
	.53 [.32, .74]	.20 [.00, .41]	.43 [.20, .67]	.72* [.58, .87]	.56 [.45, .66]	.58 [.48, .68]	.51 [.41, .61]
Number of violent charges	.09	−.07	−.05	.31**	.16	−.04	−.06
	.52 [.33, .71]	.61 [.34, .89]	.56 [.35, .76]	.64 [.43, .84]	.55 [.43, .66]	.48 [.38, .58]	.53 [.43, .63]
Number of breach of conditions charges	.09	.01	−.06	.06	.17*	−.06	−.01
	.51 [.32, .70]	.48 [.18, .78]	.63 [.42, .83]	.55 [.35, .74]	.55 [.44, .67]	.48 [.38, .58]	.50 [.40, .61]
Number of general charges	−.02	.04	.05	.06	.05	.02	−.02
	.51 [.29, .68]	.31 [.08, .55]	.57 [.37, .77]	.69* [.53, .86]	.50 [.38, .61]	.54 [.44, .64]	.51 [.41, .61]
Diversity of the criminal behaviors	.07	−.11	−.04	.30**	.08	.09	−.04
	.52 [.32, .71]	.69 [.42, .96]	.70 [.50, .90]	.79** [.68, .90]	.55 [.44, .66]	.56 [.46, .66]	.51 [.42, .61]

*Note*. Predictors in negative correlations were inverse for ROC analyses.

CSEM = child sexual exploitation material.

* *p* < .05; **p < .01.

## Discussion

This exploratory study examined behavioral and meaningful markers associated with offense-supportive cognitions commonly identified in men who engage in online sexual offending against minors. Given the limited information available about offenders’ criminogenic psychological characteristics, the study was based on in situ observations found in police files of investigations of criminal cases. We looked at a wide range of indicators and found empirical evidence of meaningful risk factors, and offending and non-offending behaviors associated with all cognitive themes.

Our findings showed associations between statements supporting the sexualization of children and meaningful risk factors commonly associated with atypical sexuality, including sexual interest in children, sex as a coping strategy, sexual preoccupation, and a history of contact sexual offending, as well as with findings from risk assessment tools (Hanson & Morton-Bourgon, 2005; Mann et al., 2010; Seto & Eke, 2015). The most prevalent category of men reporting the cognitive theme *Sexualization of children* was those who engaged in contact sexual offending against children with additional online sexual charges—in large majority CSEM-related offenses—, followed by CSEM users only. In conjunction with the association between this cognition and the number of contact sexual offending against children, our results suggest that this cognitive theme may be a general characteristic of men who engage in any form of sexual offending against children. Our findings support the hypothesis that this cognitive theme may be criminogenic as evidenced by its associations with sexual offending markers. They reinforce the idea that atypical sexual interests toward children underpin offenders’ beliefs (Bartels & Merdian, 2016; Ildeniz & Ó Ciardha, 2019), but also that this cognition may capture aspects of hypersexuality, as indicated by its association with sexual preoccupation.

Findings from the current study support previous conceptualizations of the cognitive theme *Child as partner* as a component of the construct emotional congruence with children, which is a common risk factor for sexual offending against children (Hanson & Morton-Bourgon, 2005; Mann et al., 2010; McPhail et al., 2013; Paquette & McPhail, 2020); this is evidenced by the correlation of this theme with self-reported identification with children and possession of written CSEM with a romantic component. This idea was also supported by the finding that a history of childhood abuse and some access to minors were also linked to *Child as partner*. Associations with different levels of access to minors were, however, either poor or non-existent, highlighting the need for further examination of the kinds of access to minors, especially given that opportunities facilitate sexual offending behaviors against minors (Babchishin et al., 2015; Seto, 2019; Stephens et al., 2017). There was no negative correlation between *Child as partner* and indicators of problematic relationships with adults, which is contrary to the assumption that offenders turn to children as a direct consequence of a perception that adults are untrustworthy (Ward, 2000). This is, however, coherent with previous finding showing that sexual offenders against children have separate views about children and adults, which are not dependent on each other (Paquette et al., 2014). Also, the association of this cognitive theme with indicators of pedophilia (i.e., self-reported interest in children, preference for boys, CASIC scale, and number of child pornography images possessed) further demonstrates the overlapping nature of both emotional congruence with children and pedophilia constructs (Hermann et al., 2017).

Our findings showed that the cognitive theme *Dangerous world* may be held and reported by individuals who experience difficulties with adult relationships, as it was associated with lack of or experience of problems during intimate adult relationships and self-reports of abuse during childhood. Our findings provide empirical support for the hypothesis that offline problems in adult relationships may contribute to the development of the idea that the world is a dangerous place and that adults are rejecting and cannot be trusted (Bartels & Merdian, 2016; Ildeniz & Ó Ciardha, 2019; Paquette, 2018). In our study, this cognitive theme was not related to offenders’ involvement in criminality, as indicated by its lack of association with the frequency of offending behaviors. It is possible that the small number of men reporting such cognitions may have reduced the power to detect significant effects. The significant associations between *Dangerous world* and meaningful risk factors could, however, mean that this theme may be criminogenic by nature, which would be coherent with previous findings (Paquette et al., 2019). Further examination of the relation of this theme to sexual offending markers could shed light on the role of statements supportive of the idea that the world is dangerous in sexual offending against minors.

In contrast, we found evidence supporting the idea that the cognitive theme *Entitlement* may be the cognitive component of a tendency toward being antisocial, as evidenced by longer sentences given offenders who show this trait and their involvement in non-sexual, violent criminality as well as higher levels of risk of reoffending found on assessment tools; similar associations were also found in the 2020 study by Paquette and her colleagues and further support the criminogenic nature of this cognitive theme.

Previous studies have noted that the discourse of sexual offenders sometime reflects the perception that their behaviors were uncontrollable and due to internal and external factors, including being intoxicated, having been abused during childhood, or feeling bored or lonely (Burke, 2011; Bartels & Merdian, 2016; Elliot, 2011; Paquette et al., 2014; Paquette & Cortoni, 2020a; Ward & Keenan, 1999). In the current study, we found no association between such factors and the cognitive theme *Uncontrollability*. Instead, we found a few small associations with markers associated with offenses, including scores on the CPORT scale, childhood crime history, and number of breach of conditions charges. A previous study that looked at the nature of offenders’ cognitions suggested that this theme is not by nature criminogenic and may be better understood as a combination of random justifications intended to place the blame on someone else rather than as a single criminogenic belief (Paquette et al., 2020). The links with indicators of offense found in the current study thus highlight the need for further research into the nature and role of statements supportive of the idea that offending occurs as a result of uncontrollable factors in sexual offending against minors. An additional avenue of research might be the examination of crime scene components (e.g., chat logs with the victims or other internet users, strategies employed to avoid detection or to control the victims) and their links with statements that reflect the offender’s belief that the crime occurred as a result of external factors as this might suggest strategies used (or not used) by offenders to control their crimes.

In looking at offense-supportive statements related to the use of the internet, we found that using encryption during illegal online behavior was negatively associated with the cognitive theme *Virtual is not real*. This suggests that some individuals believe that material obtained or accessed through the computer—whether sexually exploitative images of children or the identity of the person they are chatting with—is real and thus strategies to preserve their anonymity are useful, perhaps to avoid detection by law enforcement. Those who believe that internet content does not reflect reality would not see a need for such strategies. We also found that offenders who believe they are actually chatting with minors—rather than an adult or a role-playing police officer—tend to interact more frequently with minors over the internet. These findings provide new information about the role of the internet in the cognitions of online sexual offenders and when combined with our results showing negative associations between this theme and markers of sexual offending such as the STATIC99 and the overall number of child pornography charges support the hypothesis that this cognitive theme is non-criminogenic. Future research is needed to confirm or repudiate these findings. We found that those who agree that the *Internet is uncontrollable* tend to have fewer contacts with minors over online networks and fewer child luring charges. However there was a positive association between this theme and the overall number of child pornography charges. Combined results from these themes suggest specific cognitive contents for men engaging in CSEM and those exhibiting solicitation-related behaviors. Future research into the nature and criminogenic role of both internet-related cognitive themes should also consider their relation to subgroups of online sexual offenders.

### Implications for Practitioners and Researchers

The Risk-Need-Receptivity principles are now well established as an effective guideline for the treatment of sexual offenders (Hanson et al., 2009). According to this framework, treatments that focus on offenders’ criminogenic needs are more likely to reduce recidivism than those that focus on non-criminogenic needs (e.g., victim empathy; Bonta et al., 2000; Hanson & Morton-Bourgon, 2005). While it has been established that endorsing more offense-supportive cognitions is associated with sexual offending (Helmus et al., 2013), it was unclear if all the cognitive themes reported by sexual offenders against children were criminogenic in nature. In line with previous studies (e.g., Paquette et al., 2020), our findings reinforce the need for further research into specific cognitions held by those who commit sexual offenses against minors—such as ideas supportive of the sexualization of children, seeing the world and adults as dangerous, emotional identification with children, a sense of entitlement, or cognitions justifying offending behaviors by internal or external factors—to determine whether they are linked to meaningful risk factors or offending behaviors.

Looking specifically at men who engage in online sexual offending against children and adolescents clearly demonstrates that further investigation into their treatment needs is indicated (Jung, 2017) and this study provides preliminary information about the needs that should be considered. As well, an assessment scale based on behavioral markers, or at least on information available to practitioners who lack direct access to offenders, would be a great asset. Such a scale would potentially be of benefit to law enforcement agencies in prioritizing their workloads as well as to clinicians assessing the risk of reoffending for sexual offenders against minors who may be tempted to hide relevant information. Practitioners must, however, remain cautious not to draw firm conclusions on the associations between offenders’ behaviors and cognitions considering that, despite few small-to-moderate effect sizes found, many other theoretically relevant associations failed to reach even small effect sizes.

Furthermore, while it is now widely suggested that properties of the internet facilitate online sexual offending against minors (Cooper et al., 2000; Quayle & Taylor, 2003; Seto, 2013), this hypothesis has not been empirically established. Although there have been few attempts to establish the role of the internet in online sexual offenses, much more remains to be done. Paquette and Cortoni (2020b), using a psychometric measure, examined whether self-reported perceptions of anonymity in online social media were associated with CSEM-related and child sexual solicitation-related offending and found no significant association. In the CPORT development study, Eke and Seto (2016) tested whether the use of a public computer was predictive of sexual recidivism but failed to establish such a link. They recommend examining links between online sexual offending behaviors and activities that increase secrecy, including the use of encryption, proxy servers, TOR browsers, or online networks that provide specific information about how to avoid detection and see that computers are secure (Eke & Seto, 2016). We found some associations between internet properties and cognitive themes; the next step would be to examine the criminogenic nature of both statements and properties. Some variables that were not tested in the current study might be worth investigating to determine if they are associated with offense-supportive statements and online sexual offending behaviors. For instance, evidence of internet searches for information on how to avoid police detection could indicate that virtual content and activities are perceived as real. Evidence of protection software or activation of controlling parameters to ensure avoiding visiting illegal websites may be associated with the idea that the *Internet is uncontrollable* and that one should act accordingly to avoid legal problems.

From a law enforcement perspective, a better understanding of those suspected of consuming child sexual exploitation material or engaging in online sexually solicitative communications with adolescents would be helpful in police interrogations. There is evidence that certain personality or individual characteristics and positive relationships with the suspect increase the likelihood of obtaining a confession during police interrogations (Beauregard et al., 2010; Deslauriers-Varin et al., 2018, 2019). Our findings, although preliminary, provide some information about what is in suspects’ mind and will make it possible to adapt interrogation approaches accordingly.

## Limitations and Conclusion

Limits to this study should be noted. First, while our goal was to provide practitioners with behavioral indicators of offense-supportive cognitions, the police dataset that formed the basis for our data had limitations. While the case files contained relevant information and additional material was obtained during interviews with police, some information that would have been helpful was not consistently available as police and researchers have very different goals. Consequently, we could only code for the presence of the variables among the suspects, not their absence—at least not systematically. For instance, while some offenders disclosed a history of childhood abuse, victimization was not consistently discussed during interrogation so some individuals who had been victimized during childhood may not have disclosed this information. Other themes, such as sexual interest in children, were more consistently discussed. As well, the duration of police interrogations varied considerably (from one to up to ten hours), which may have influenced the amount of information available.

Second, the small sample size of specific subgroups of offenders meant that we were unable to conduct individual analyses for men with CSEM-related offenses, online solicitation offenses involving minors, and contact sexual offenses. Given the heterogeneity those in these subgroups, especially between CSEM users who tend to target younger children and those who tend to target teenagers (Wolak et al., 2004; Wurtele, 2017), separate analyses might have provided a better understanding of the distinctive factors associated with each type of online sexual offense against minors. Future research should consider looking at these subgroups of online sexual offenders separately.

In conclusion, this exploratory study helped identify behavioral and meaningful risk markers that could suggest offense-supportive cognitions among men who engage in exploitative sexual behaviors against minors, online, offline, or both. In a perspective of looking at the role of cognitions in sexual offending in future studies, this study has contributed to refining the initial conceptualization of offenders’ cognitive themes by examining their potential correlates. We found that markers of pedophilia were associated with *Sexualization of children*, markers of emotional congruence with children were associated with *Child as partner*, markers of intimate relation problems were associated with *Dangerous world*, and markers of criminal involvement were associated with *Entitlement* and *Uncontrollability*, although to a lesser extent. Finally, the use of encryption during offending behavior on the internet was found to be negatively associated with *Virtual is not real*, while the level of access to minors using virtual networks was found to be linked to the *Internet is uncontrollable*. Findings from this study need to be replicated using larger samples but could ultimately be helpful in the development of assessment tools that would be of use to both clinicians and law enforcement agencies.

## Supplemental Material

sj-pdf-1-ijo-10.1177_0306624X211013523 – Supplemental material for A Peek into Their Mind? An Exploration of Links Between Offense-Supportive Statements and Behaviors among Men Who Sexually Exploit Children and Adolescents Online Click here for additional data file. Supplemental material, sj-pdf-1-ijo-10.1177_0306624X211013523 for A Peek into Their Mind? An Exploration of Links Between Offense-Supportive Statements and Behaviors among Men Who Sexually Exploit Children and Adolescents Online by Sarah Paquette and Francis Fortin in International Journal of Offender Therapy and Comparative Criminology
