# Supplementary material for: A Peek into Their Mind? An Exploration of Links Between Offense-Supportive Statements and Behaviors among Men Who Sexually Exploit Children and Adolescents Online
Source: Int J Offender Ther Comp Criminol. 2021 May 6;67(6-7):591–617. doi: 10.1177/0306624X211013523 (PMC10052436; doi:10.1177/0306624X211013523)
Supplement: sj-pdf-1-ijo-10.1177_0306624X211013523 – Supplemental material for A Peek into Their Mind? An Exploration of Links Between Offense-Supportive Statements and Behaviors among Men Who Sexually Exploit Children and Adolescents Online [file sj-pdf-1-ijo-10.1177_0306624X211013523.pdf]

Supplemental Material

Table S1. Variable Codification and Inter-Rater Agreement

Cognitive Themes	Definition	Examples to support codification	Frequence or mean (S.D., range)	Inter-rater agreement<br>*n* = 2 raters<br>*n* = 9 cases
Child as sexual being	Statements reflecting the perception that children are sexual beings capable of providing free and informed consent with regard to sex or that their sexual maturity is equal to that of adults.	"Children are motivated toward sexual pleasure and want to have relationships with adults." ; "Children have a sexual maturity equal to that of adults."; "Children can give free and informed consent."; "I touched him because he was still stuck on me."; "With this child it was different, he was willing."; "She was the one who wanted to get naked in front of her webcam."; "I proposed meeting her but I would never have forced her."	19.7%	88.89%
Nature of harm	Statements supporting the idea that sexual abuse does not cause harm to children but, on the contrary, can be beneficial. It also refers to the idea that there are various degrees of harm caused by sex abuse.	"I did not think it harmed the child because my own abuse did not cause me any harm."; "I did not hit him, so it's not really an assault."; "Yes I did chatted about sex with children, but I have not abused anyone."	20.4%	77.78%
Child as partner	Statements supporting the idea that children are friends, lovers, or intimate life partners. This does not concern sexual interactions between the offender and his victim but instead concerns relationships perceived by the offender as based on friendship, affection, and intimacy. Offender may perceive himself as	"This relationship had nothing to do with sex, it was a way of giving and receiving affection."; "In my head, I was also a child."; "In my head, I was part of the gang."; "I chat online with children to make friends."	8.0%	77.78%

	equal to children, in both social and emotional aspects.			
Dangerous world	Statements supporting the idea that the world is a hostile place, that individuals exploit others and live according to their own interests. Adults are perceived as threatening and untrustworthy. Because they perceive adults negatively, sex offenders engage in relations with children. They perceive them as being trustworthy and safe.	“I avenged myself on my ex-spouse by sexually assaulting his child.”; “You cannot trust adults.”; “I’m not looking for a relationship with women because I think I'm not good enough for them. They are too beautiful and intelligent.”; “Women reject me all the time.”	7.3%	88.89%
Entitlement	Statements supporting the idea that some people would be superior to others. Because of a privileged status, they perceived they are entitled to satisfy their needs over others, and expect these acts to be accepted by all, especially children.	“Sex with children is different from that with adults. With the children, I decide what to do.”; “I have the right to have sex with my daughter if my spouse does not want to have it with me.”; “As a teacher, I was giving her sex education.”; “The sex conversations, it was just a power trip.”	7.3%	88.89%
Uncontrollability	Statements reflecting the idea that sexual abuse occurs due to a lack of control over sexual emotions, impulses and behaviors. Responsibility for sexual crimes tend to be rejected on victims, external factors or negative events.	“It was not my fault, I was under the influence of drugs when I touched it.”; “It is her mother's fault. She asked me to go and show her how to wash in the shower.”; “My sex drive is too strong for me to stop ...”	26.3%	88.89%
Virtual is not real	Statements reflecting the idea that the Internet does not represent reality, that one cannot know its content, that all that is written is a lie or a joke. It is also the idea according to which one cannot know with whom we chat.	“She said she was 13, but in reality it must be a 50-year-old man.”; “The conversations were role plays.”; “I look at child pornography but I have no sexual interest toward children in real life.”	40.1%	77.78%
Internet is uncontrollable	Statements supporting the idea that the Internet is uncontrollable. Online sexual offences are justified by the fact that on the Internet, contents are *too* accessible. The Internet is	“No offense would have been committed if the Internet did not exist.”; “With the Internet, it's too easy to find child pornography.”; “There were pop-ups that appeared on my computer showing illegal contents.”	36.5%	100%

	perceived as a facilitator, even a temptation to commit sexual crimes. Without the Internet, the crimes would not have been committed.			
**Meaningful Risk Factors**				
Sexual interest towards children	Evidence or self-admission of sexual interest towards children, or of a previous diagnosis of pedophilia.		38%	77.78%
Sexual preoccupation	Self-admission about being concerned or obsessed with sex. This may refer to sexual thoughts, fantasies, masturbation, use of pornography, or other behaviors.	“I try to control myself, but I always go back to the Internet to watch child pornography images ... I think about it every day.”	20.4%	88.89%
Sexual preference for boys	Self-admission of a sexual preference for male children		15.3%	77.78%
Emotional congruence with children	Self-admission about self-identifying on affective, emotional or cognitive level with children	“In my head, I am still a child. I feel they understand me better than adults understand me.”	11.7%	88.89%
History of childhood abuse	Evidence or self-admission of physical, sexual or psychological abuse in family or school environment during childhood or adolescence.	Physical abuse is a deliberate use of force against a person without their consent. It can cause physical pain or injury that may be permanent. Examples include pushing or shoving, hitting, slapping or kicking, pinching or punching, strangling, stabbing, shooting, throwing objects at someone, causing burns, holding someone while someone else assaults them, locking someone in a room or tying them up, killing someone.<br><br>Sexual abuse includes sexual touching, sexual activity, or intercourse.<br><br>Psychological abuse refers to the use of words or actions	29.9%	75.93%

		to control, frighten, isolate or undermine a person's dignity. Examples include threatening, belittling, name-calling or insulting, constantly yelling at or criticizing someone, blocking access to family or friends, destroying property, hurting pets or threatening to do so, intimidating or humiliating (including on the Internet).		
Sex as coping strategy	Self-admission of masturbating, using any form of pornography or sexually fantasizing whenever experiencing difficult, stressful or negative situations.		.18, (.53, 0-3)	92.59%
Relational difficulties	Self-admission of difficulties in intimate relationships with adults.		26.3%	77.78%
Never been in an intimate relationship	Self-admission of having never been in an intimate relationship with an adult for more than 2 years.		22.6%	66.67%
Length of longest relationship	Length (in months) of the longest intimate relationship.		127.43 (139.90, 0-612)	r = .91
**Non-Offending Markers and Behaviors**				
Access to minors via volunteering	Has or had an unpaid role in which it is expected that there will be unsupervised access to children - creates an opportunity to commit sexual offenses.	Scout leader, coach for a sports team	15.3%	77.78%
Access to minors via work	Has or had a job in which it is expected that there will be unsupervised access to children - creates an opportunity to commit offenses.	Teacher	10.2%	88.89%
Access to minors via family	Lives with a child at the time of his arrest for the most recent offense.		45.3%	77.78%

Access to minors via online networks	Maintenance of online communications / virtual contacts with children.	With his daughter’s friend, with a child met on Instagram	48.9%	100%
Number of modes of access	Composite of access to minors via volunteering, work, family, and virtual through online networks.		1.61 (.90, 0-5)	86.11%
Encryption	Use of encryption software or passwords.	VeraCrypt	12.4%	77.78%
Hidden identity software	Software or tools were used to hide the identity of the offender.	Tor, Proxys, PGP, etc.	1.5%	100%
Public computer	Use of a public computer or one accessible by others to commit sexual offenses.	Internet coffee shop, library, McDonald’s	14.6%	66.67%
Number of anonymity strategies	Composite of encryption, hidden identify software, and public computers.		.40 (.59, 0-2)	81.48%
Feeling bored	Self-admission of feeling bored, referring to feelings of weariness, emptiness, melancholy, lack of interest, feelings of monotony when engaging in sexual offences.		10.2%	66.67%
Feeling lonely	Self-admission of isolation, referring to feelings of loneliness, with few or no significant people in his life – often occurs when engaging in sexual offences.		26.3%	77.78%
**Sexual and Non-Sexual Offending Behaviors**				
Number of child victims	Total number of known child victims - either from online solicitation or contact sexual offending.		n = 39, 2.51 (2.29, 1-12)	r = .98
Number of male child victims	Total number of known male child victims - either from online solicitation or contact sexual offending.		.92 (2.10, 0-12)	r = .99

Number of child pornography images	Total number of child pornography images and videos found on offender's computers during police seizure.	Includes images from category 1, child pornography, as defined by the Canadian criminal code, article 163.1. Includes images from category 2, child nudity, as defined as sexual photos presenting children completely nude or partially dressed. Although it does not exactly fit the definition of child pornography, these images are aggravating and are used by law enforcement to assist the court in assessing sexual interest.	20,330.99 (118,555.94, 0-1,071,226)	r = .98
Number of contact sexual charges	Lifetime number of contact sexual offenses.	Includes crimes against both adults and children.	.71 (1.77, 0-11)	n/a
Number of child pornography charges	Lifetime number of child pornography charges.	Includes accessing, possessing, distributing, or producing child pornography.	2.82 (4.01, 0-38)	n/a
Number of child luring charges	Lifetime number of child luring charges.		1.79 (4.42, 0-34)	n/a
Masturbating while viewing minors	Evidence or self-admission of masturbating while viewing child pornography or during a webcam session with a minor.		24.8%	100%
Written CSEM with romantic component	Evidence or self-admission of having accessed written child pornography with an affective / emotional component.		3.6%	100%
Substance abuse	Evidence or self-admission of problems with the use of drugs or alcohol.		19.0%	88.89%

Childhood crime history	Self-admission of having engaged in criminal behavior before the age of 18.		5.8%	66.67%
Length of index crime sentence	Number of days of sentence for index crime.		450.29 (400.77, 0-2373)	n/a
Number of violent charges	Lifetime number of violent charges.	Includes threatening to, attempting to, or violating the physical integrity of a person, such as assault, murder, threats, robbery, extortion, and using or brandishing a weapon.	.33 (1.35, 0-14)	n/a
Number of breach of conditions charges	Lifetime number of breach of conditions charges.	Illegal liberty, omissions, etc.	1.14 (2.38, 0-13)	n/a
Number of general charges	Lifetime number of general charges.	Includes all non-sexual and non-violent criminal offenses, such as theft, mischief, use of drugs, etc.	1.71 (4.03, 0-19)	n/a
Diversity of criminal behaviors	Composite of sexual, violent, and general crimes.		1.69 (.67, 1-3)	n/a

n/a = not applicable. Information taken from official sources has not been subjected to inter-rater agreement assessment.

CSEM = child sexual exploitation material
